# GPR37 expression as a prognostic marker in gliomas: a bioinformatics-based analysis

**DOI:** 10.18632/aging.205063

**Published:** 2023-10-13

**Authors:** Kairong Liang, Zhaoxiong Guo, Shizhen Zhang, Danmin Chen, Renheng Zou, Yuhao Weng, Chengxiang Peng, Zhichao Xu, Jingbai Zhang, Xiaorui Liu, Xiao Pang, Yunxiang Ji, Degui Liao, Miaoling Lai, Huaidong Peng, Yanbin Ke, Zhaotao Wang, Yezhong Wang

**Affiliations:** 1 Institute of Neuroscience, Department of Neurosurgery, The Second Affiliated Hospital of Guangzhou Medical University, Guangzhou 510260, China; 2 Science and Technology Innovation Center, Institute of Clinical Pharmacology, Guangzhou University of Chinese Medicine, Guangzhou 510405, China; 3 Department of Pharmacy, The Second Affiliated Hospital of Guangzhou Medical University, Guangzhou 510260, China; 4 Department of Pharmacy, Affiliated Cancer Hospital and Institute of Guangzhou Medical University, Guangzhou 510095, China

**Keywords:** glioma, *GPR37*, prognosis, immune infiltration, M2 macrophages

## Abstract

Background: Gliomas are the most frequently diagnosed primary brain tumors, and are associated with multiple molecular aberrations during their development and progression. *GPR37* is an orphan G protein-coupled receptor (GPCR) that is implicated in different physiological pathways in the brain, and has been linked to various malignancies. The aim of this study was to explore the relationship between *GPR37* gene expression and the clinicopathological factors, patient prognosis, tumor-infiltrating immune cell signature GSEA and methylation levels in glioma.

Methods: We explored the diagnostic value, clinical relevance, and molecular function of GPR37 in glioma using TCGA, STRING, cBioPortal, Tumor Immunity Estimation Resource (TIMER) database and MethSurv databases. Besides, the "ssGSEA" algorithm was conducted to estimate immune cells infiltration abundance, with 'ggplot2' package visualizing the results. Immunohistochemical staining of clinical samples were used to verify the speculations of bioinformatics analysis.

Results: *GPR37* expression was significantly higher in the glioma tissues compared to the normal brain tissues, and was linked to poor prognosis. Functional annotation of *GPR37* showed enrichment of ether lipid metabolism, fat digestion and absorption, and histidine metabolism. In addition, GSEA showed that *GPR37* was positively correlated to the positive regulation of macrophage derived foam cell differentiation, negative regulation of T cell receptor signaling pathway, neuroactive ligand receptor interaction, calcium signaling pathway, and negatively associated with immunoglobulin complex, immunoglobulin complex circulating, ribosome and spliceosome mediated by circulating immunoglobulin etc. TIMER2.0 and ssGSEA showed that *GPR37* expression was significantly associated with the infiltration of T cells, CD8 T cell, eosinophils, macrophages, neutrophils, NK CD56^dim^ cells, NK cells, plasmacytoid DCs (pDCs), T helper cells and T effector memory (Tem) cells. In addition, high *GPR37* expression was positively correlated with increased infiltration of M2 macrophages, which in turn was associated with poor prognosis. Furthermore, *GPR37* was positively correlated with various immune checkpoints (ICPs). Finally, hypomethylation of the *GPR37* promoter was associated with its high expression levels and poor prognosis in glioma.

Conclusion: *GPR37* had diagnostic and prognostic value in glioma. The possible biological mechanisms of *GPR37* provide novel insights into the clinical diagnosis and treatment of glioma.

## INTRODUCTION

Adult-type diffuse gliomas are the most frequent invasive primary central nervous system (CNS) tumor and is associated with a high fatality rate [1]. According to the fifth edition of WHO Central Nervous System Tumor Classification (CNS5 WHO), adult-type diffuse gliomas are divided into three categories: glioblastoma, IDH wild type (grade 4); Oligodendroglioma, IDH mutant, *1p/19q* co-deletion, (grade 2 and 3); Astrocytoma, IDH mutant (grade 2, 3 and 4) [2]. Surgery, radiotherapy and chemotherapy are the primary treatment strategies against glioma, and have achieved only limited improvements in patient prognosis [3]. In CNS5 WHO, some tumor molecular features (such as IDH1/2 mutation, TERT promoter mutation, EGFR amplification, H3 mutation, +7/−10, *1p/19q*-codeletion, *CDKN2A/B* homozygous deletion, etc.,) that significantly impact on the prognosis of glioma have been further integrated and directly included in the diagnosis [4]. Nevertheless, it is still necessary to discover novel markers of glioma to improve diagnosis and predict prognosis with greater accuracy.

*GPR37* is a member of G-protein-coupled receptors (GPCRs), also known as Parkinson’s related endothelin like receptor (Pael-R). It is highly expressed in the brain and is related to neurological diseases such as Parkinson’s disease and autism [5]. Knocking down *GPR37* in lung adenocarcinoma (LUAD) cells inhibited the malignant behavior [6], whereas elevated *GPR37* in gastric cancer cells is linked to peritoneal metastases and poor prognosis [7]. In addition, Wang et al. and Huang et al. established *GPR37* as a potential prognostic biomarker for lung adenocarcinoma and human multiple myeloma [8, 9]. Up-regulation of *GPR37* in the U251 glioma cell line accelerated cell cycle progression, activate AKT pathway and promote proliferation [10]. However, there is little conclusive data regarding the involvement of *GPR37* in the genesis and progression of glioma.

To that end, we investigated the prognostic significance and putative biological functions of *GPR37* in glioma using bioinformatics.

## MATERIALS AND METHODS

### Expression analysis and survival analysis of *GPR37*

Data from GEPIA2 (http://gepia2.cancer-pku.cn/#index) was used to validate *GPR37* mRNA expression levels in cancer and normal tissues [11]. The differential expression of *GPR37* between the glioma and normal tissues was analyzed by combining data from the GTEx (http://commonfund.nih.gov/GTEx) and The Cancer Genome Atlas (TCGA) (https://portal.gdc.cancer.gov/repository), which had been uniformly processed by the Toil process in UCSC Xena (https://xenabrowser.net/datapages/) [12–14]. Datasets of glioma patients, including both genetic and clinical information, were retrieved from TCGA database and plotted using the R ggplot2 (version: 3.6.3) package.

Kaplan-Meier curves for the overall survival (OS) were plotted based on TCGA data, and compared using Cox regression. The R survival (version: 3.2-10) and survminer (version: 0.4.9) packages were used for statistical analysis and visualization.

Immunohistochemical data of *GPR37* the protein expression and distribution were analyzed in the HPA database (https://www.proteinatlas.org/) [15].

### Patients and sample

Specimens of tumor and adjacent tissues were collected from 38 patients in the Second Affiliated Hospital of Guangzhou Medical University, who had undergone curative surgery from 2020 to 2022 in our hospital, which was approved by Institutional Ethics Committee in the Second Affiliated Hospital of Guangzhou Medical University. 38 patients’ tumor tissues were used for immunohistochemistry. Written informed consents were acquired from each patient relying on guidelines of the Declaration of Helsinki.

### Immunohistochemistry (IHC)

Tissue sections were deparaffinised, soaked in TrisEDTA buffer (pH 9.0) boiled in a microwave and then incubated with antibodies against *GPR37* (1:250; ab218134, Abcam) at 4°C for 12 h. The next day, slides were washed, stained with secondary antibodies and 3, 3′-diaminobenzidine, counterstained with hematoxylin, dehydrated and mounted. The sections were reviewed and scored independently by two observers. The results of immunohistochemistry of *GPR37* between the different glioma was analyzed by Image Pro Plus image analysis software [16], take the average optical density (AOD) as the measurement index and plotted using the R ggplot2 (version: 3.6.3) package [17].

### Univariate and multivariate cox regression analysis

TCGA data were combined and plotted in a matrix. The impact of *GPR37* expression and other clinicopathological parameters (grade, histological type, *CDKN2A/B* homozygous deletion, age, gender and primary therapy outcome) on the OS and DSS was evaluated using univariate and multivariate cox analysis, with *P* value < 0.05 as the cut-off criterion. The R package ‘forestplot,’ was used to calculate the *P* value, HR and 95% CI of each variable.

### Gene set enrichment analysis and co-expressed genes

The gene co-expressed with *GPR37* were obtained using the LinkFinder module of LinkedOmics (http://www.linkedomics.org/), and a heat map of the top 50 positively or negatively correlated genes was generated [18]. The genes and proteins that physically interact with *GPR37* were identified using the STRING database (https://string-db.org) [19], and a protein-protein interaction (PPI) network was built with a total score of > 0.7 (high confidence) and visualized using Cytoscape [20]. The glioma samples of TCGA database were then separated into the *GPR37*^high^ and *GPR37*^low^ groups, and the differentially expressed genes (DEGs) between the groups were identified using the DESeq2 R (version: 1.26.0) package with *P* < 0.05 and |log FC|≥1.0 as the thresholds [21]. The hub genes were functionally annotated using Gene Ontology (GO) keywords (biological process, cellular component and molecular function categories) and Kyoto Encyclopedia of Genes and Genomes (KEGG) pathways [22, 23].

The Clusterprofiler program was used to perform gene set enrichment analysis (GSEA) to identify biological pathways that differed significantly between the *GPR37*^high^ and *GPR37*^low^ groups [24, 25]. Studies were run in the MSigDB database (https://www.gsea-msigdb.org/gsea/msigdb/collections.jsp#C2) with a number of size 3 and 10000 simulations [26]. Genes with false discovery rate (FDR) < 0.25 and *p*_adjust_ < 0.05 were considered statistically significant.

### Tumor infiltration analysis

The single-sample GSEA (ssGSEA) was used to quantify the tumor infiltration of 24 immune cell types based on TCGA data using the R GSVA package [27, 28]. The gene panels for each immune cell type were selected as per a recent report. The correlation of *GPR37* expression with the infiltration of eosinophils, macrophages, NK cells, neutrophils and T cells was analyzed. OS was examined as a function of *GPR37* expression, M2 macrophage and cancer-associated fibroblasts (CAFs) in the TIMER2.0 (http://timer.cistrome.org) database [29].

### DNA methylation analysis

The relationship between DNA methylation and *GPR37* expression was investigated using Pearson correlation analysis. Correlation coefficients (R) and Benjamin–Hochberg-adjusted *P*-values for different methylation sites were obtained. *GPR37* methylation and the Kaplan–Meier-based correlation between *GPR37* hyper/hypomethylation and OS were visualized using the MethSurv (https://biit.cs.ut.ee/methsurv) program [30].

### Availability of data and materials

All of the data utilized in this study came from publicly accessible databases. The databases that were used throughout the investigation are listed below. Gene Expression Profiling Interactive Analysis 2 database (GEPIA2, http://gepia2.cancer-pku.cn/#index), Genotype-Tissue Expression Project (GTEx, http://commonfund.nih.gov/GTEx), The Cancer Genome Atlas (TCGA, https://portal.gdc.cancer.gov/repository), XENA platform (UCSC Xena, https://xenabrowser.net/datapages/), Human Protein Atlas (https://www.proteinatlas.org/), LinkedOmics database (http://www.linkedomics.org/), STRING database (https://string-db.org), Molecular Signatures database (MSigDB, https://www.gsea-msigdb.org/gsea/msigdb/collections.jsp#C2), Tumor Immune Estimation Resource 2.0 database (TIMER2.0, http://timer.cistrome.org), MethSurv database (https://biit.cs.ut.ee/methsurv).

## RESULTS

### *GPR37* is overexpressed in glioma and associated with clinicopathological factors

**Figure 1:**
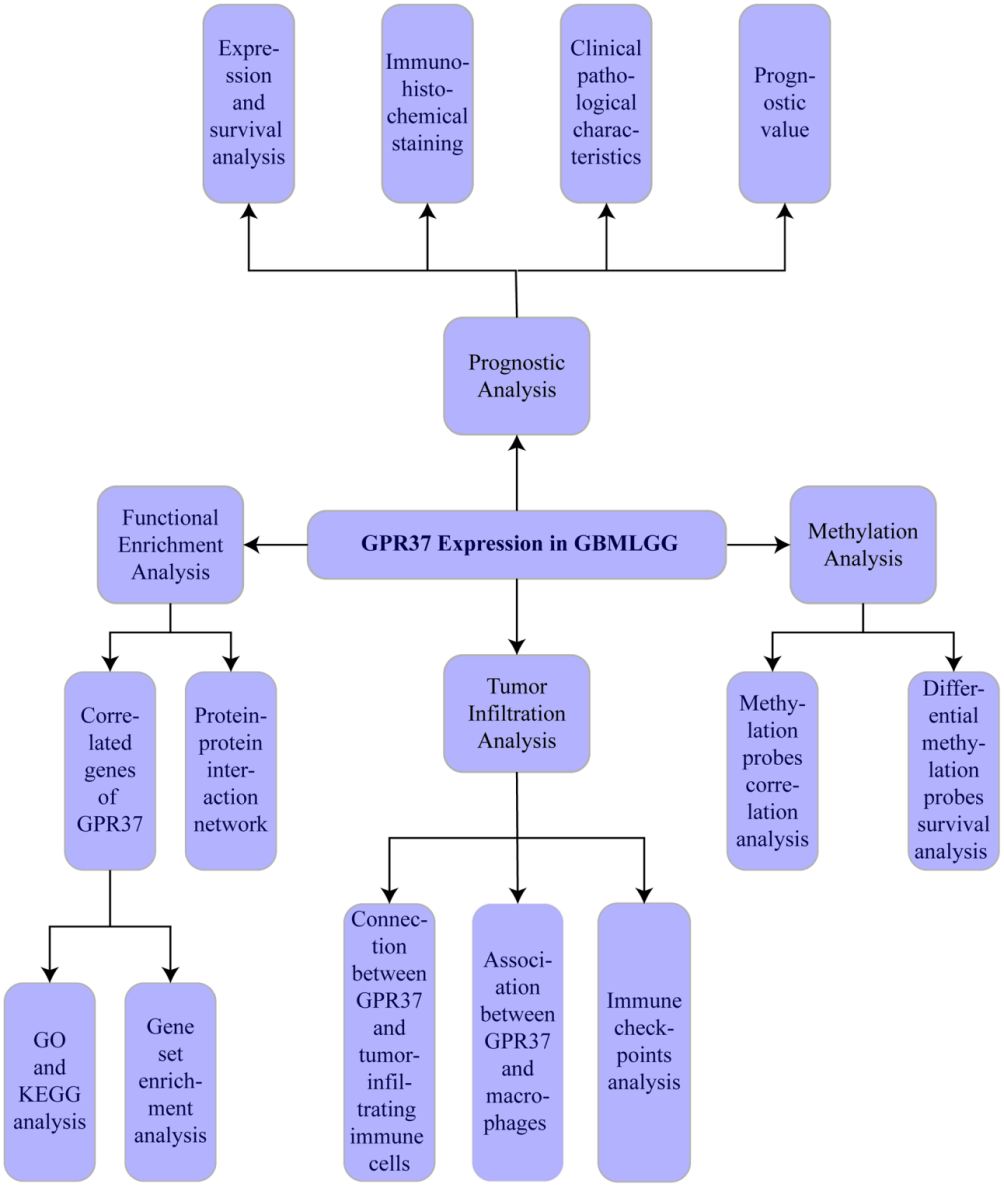
Flow chart of the study.

**Figure 2:**
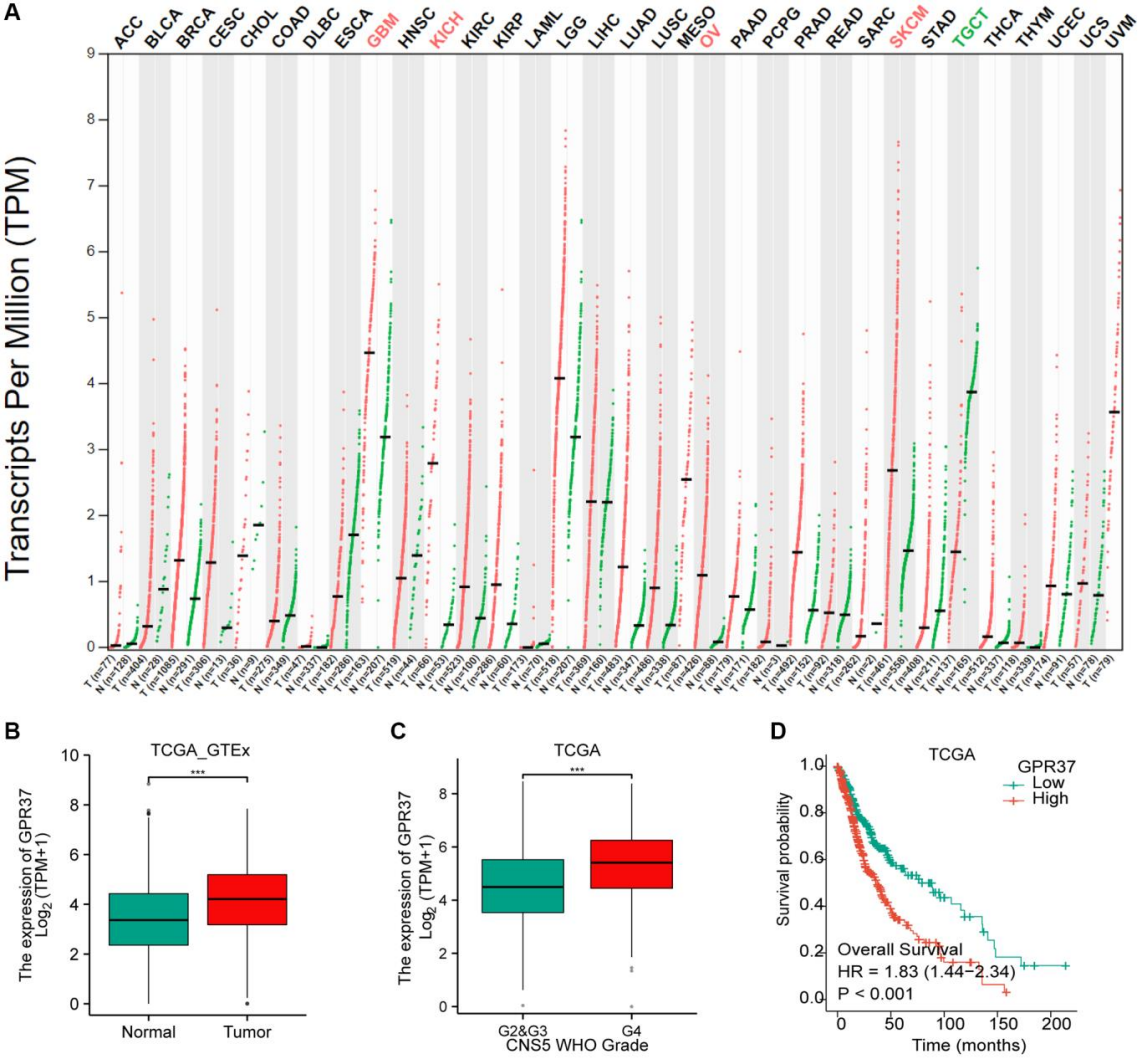
**Differential *GPR37* expression in glioma and prognostic relevance.** (**A**) Pan-cancer *GPR37* mRNA levels. (**B**) *GPR37* expression across glioma samples and normal tissues. (**C**) Survival curves of *GPR37*^high^ and *GPR37*^low^ glioma patients in TCGA dataset (**C**, **D**). Abbreviations: OS: overall survival; LGG: low grade glioma.

**Figure 3:**
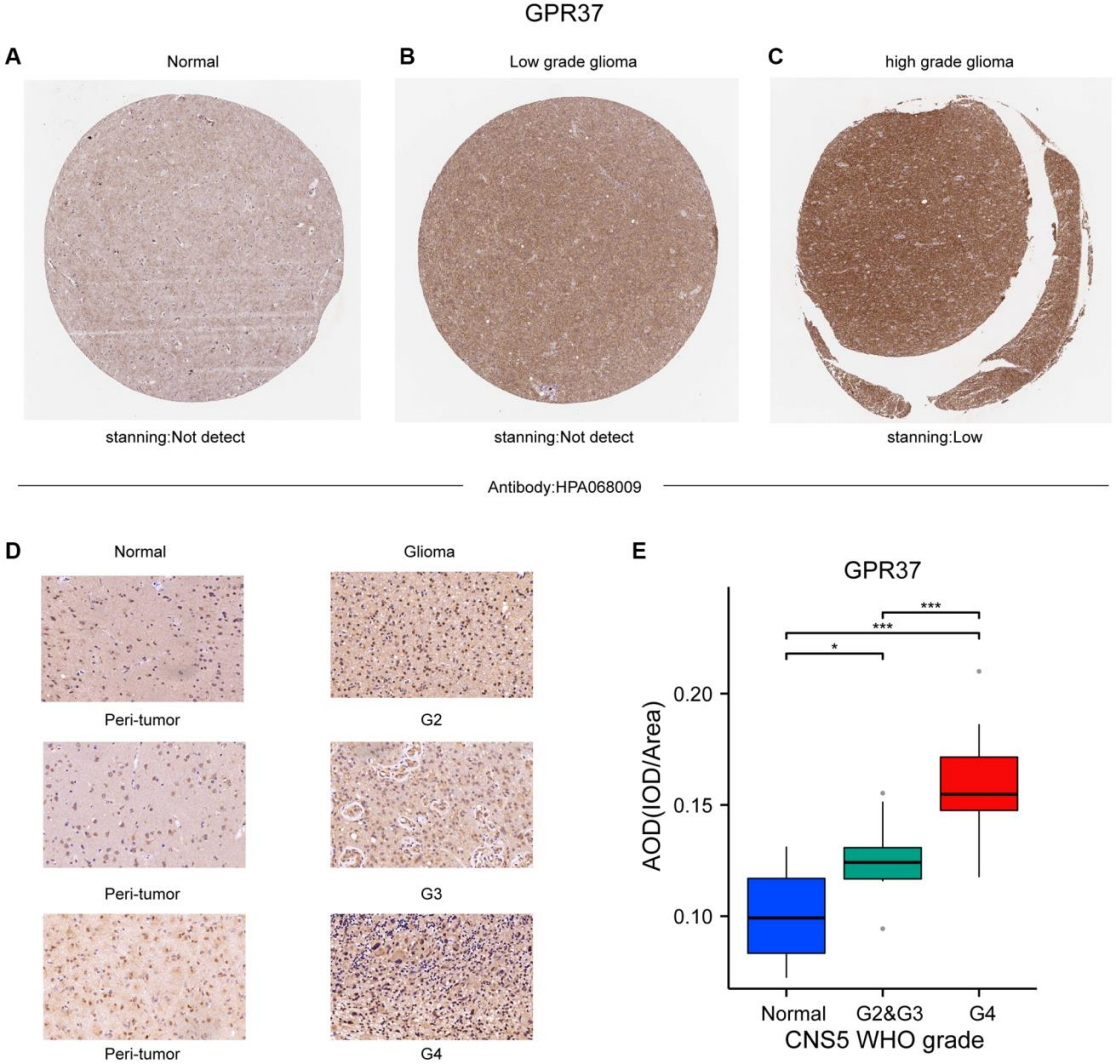
(**A**–**C**) Representative IHC images from the Human Protein Atlas showing *in situ GPR37* protein expression in glioma tissues. (**D**, **E**) GPR37 expression in different grades of glioma was statistically analyzed in 38 gliomas using normal tissues adjacent to the tumor as the control group.

The flow chart of the study is shown in Figure 1. The classification of histological type and grade of all glioma samples (listed in Supplementary Table 1) are consistent with the recommendations of CNS5 WHO [31]. *GPR37* expression levels were analyzed in pan-tumor tissues and their normal counterparts (Figure 2A). As shown in Figure 2B, *GPR37* was significantly up-regulated in glioma compared to normal brain tissues (Figure 2B). Furthermore, *GPR37* expression was higher in CNS WHO grade 4 tumors relative to the CNS WHO grade 2 & 3 tumors (Figure 2C). Consistent with this, overexpression of *GPR37* was linked to poor prognosis in glioma patients in terms of the likelihood of overall survival (OS) (Figure 2D, *P* < 0.001). In addition, immunohistochemical analysis was applied to observe the distribution and protein levels of *GPR37*. The clinicopathological characteristics of 38 patients are shown in Supplementary Table 2. With normal tissues adjacent to the tumor as the control group, the *GPR37* expression in different grade of glioma were statistically analyzed in 38 samples. As shown in Figure 3A–3C, *GPR37* was expressed in neurons and glia and exhibited more elevated expression levels in GBM tissues. Immunohistochemical staining of clinical 38 samples also confirmed that the different level of *GPR37* expression in tumor tissues were in CNS WHO grade 2, 3 and 4 of glioma (Figure 3D, 3E).

We further analyzed the data from TCGA databases to explore a possible link between *GPR37* and clinicopathological characteristics of glioma patients. The patients were divided into *GPR37*^high^ (*n* = 353) and *GPR37*^low^ (*n* = 352) groups based on the median expression value. As shown in Table 1, elevated *GPR37* in glioma was significantly associated with the CNS WHO grade, histological type,*1p/19q* codeletion, the IDH status, *CDKN2A/B* homozygous deletion, age and primary therapy outcome (*P* < 0.05), while no significant correlation was seen with gender (*P* > 0.05). Moreover, univariate logistic regression analysis (Table 2) showed a significant correlation of *GPR37* mRNA expression with CNS WHO grade (G4 vs. G2 & G3, OR = 0.380, 95% CI (0.069–0.692), *P* < 0.001), histological type (Glioblastoma, IDH wildtype vs. Oligodendroglioma, IDH mutation, *1p/19q*-codel vs. Astroctyoma, IDH mutation, OR = 0.397, 95% CI (0.040–0.754), *P* < 0.001), *CDKN2A/B* homozygous deletion (non-homdel vs. homdel, OR = 1.865, 95% CI (1.495–2.234), *P* < 0.001), age (≤60 vs. >60, OR = 1.466, 95% CI (1.095–1.836), *P* < 0.001).

**Table 1:** GPR37 mRNA expression and clinicopathological variables of glioma.

**Characteristics**	**Low expression of *GPR37***	**High expression of *GPR37***	***P* value**
*n*	353	352	
Grade, *n* (%)			
G4	103 (14.8%)	182 (26.1%)	<0.001
G2 & G3	247 (35.4%)	166 (23.8%)	
Histology, *n* (%)			
Glioblastoma, IDH WT	81 (11.7%)	166 (24%)	<0.001
Oligodendroglioma, IDH mut, *1p/19q* codel	115 (16.6%)	57 (8.2%)	
Astroctyoma, IDH mut	150 (21.7%)	122 (17.7%)		
*CDKN2A/B* homozygous deletion, *n* (%)			
Non-homdel	296 (42%)	259 (36.7%)	<0.001
Homdel	57 (8.1%)	93 (13.2%)	
IDH, *n* (%)			
WT	81 (11.7%)	166 (24%)	<0.001
Mutant	265 (38.4%)	179 (25.9%)	
*1p/19q*, *n* (%)			
Non-codel	233 (33.6%)	289 (41.6%)	<0.001
Codel	115 (16.6%)	57 (8.2%)	
Gender, *n* (%)			
Male	202 (28.9%)	199 (28.5%)	0.922
Female	149 (21.3%)	149 (21.3%)	
Age, *n* (%)			
≤60	290 (41.5%)	266 (38.1%)	0.043
>60	61 (8.7%)	82 (11.7%)	
Primary therapy outcom, *n* (%)			
CR	67 (14.4%)	73 (15.7%)	0.037
SD	81 (17.4%)	67 (14.4%)	
PD	51 (11%)	61 (13.1%)	
PR	43 (9.2%)	22 (4.7%)	

**Table 2:** GPR37 expression correlated with clinicopathological characteristics.

**Characteristics**	**Total (*N*)**	**Odds Ratio (95% CI)**	***P* value**
CNS WHO grade (G4 vs. G2 & G3)	698		
G4	285	Reference	
G2 & G3	413	0.380 (0.069–0.692)	**<0.001**
Histological Type	691		
Glioblastoma, IDH wildtype	247	Reference	
Oligodendroglioma, IDH mutation, *1p/19q*-codel	172	0.242 (−0.172–0.656)	**<0.001**
Astroctyoma, IDH mutation	272	0.397 (0.040–0.754)	**<0.001**
*CDKN2A/B* homozygous deletion	705		
Non-homdel	555	Reference	
Homdel	150	1.865 (1.495–2.234)	**<0.001**
Age	699		
≤60	556	Reference	
>60	143	1.466 (1.095–1.836)	**0.043**
Gender	699		
Male	401	Reference	
Female	298	1.015 (0.715–1.315)	0.922
Primary therapy outcome	465		
PR & CR	205	Reference	
PD & SD	260	1.123 (0.756–1.490)	0.536

### *GPR37* expression is an independent prognostic factor in glioma

**Figure 4:**
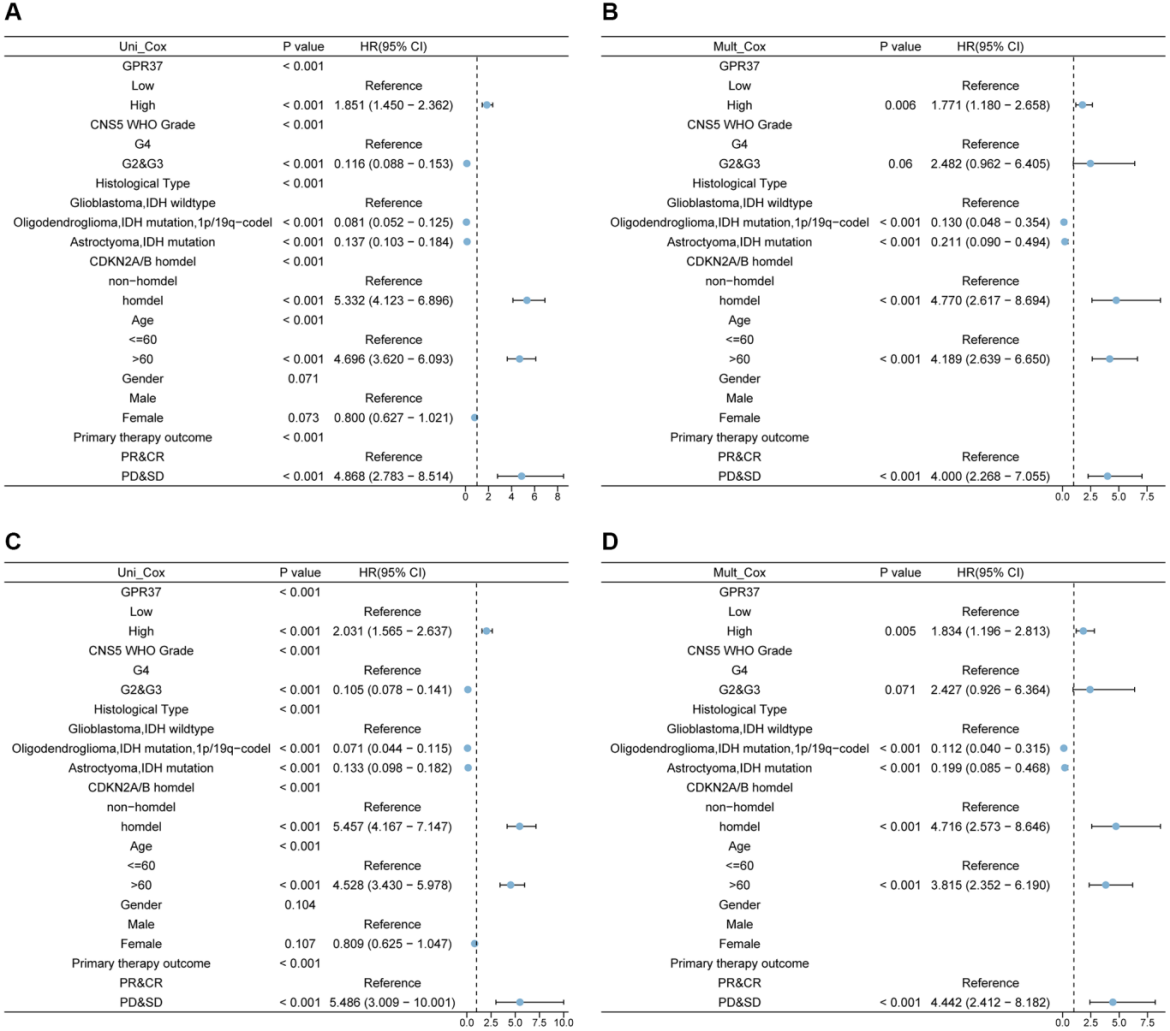
Univariate and multivariate Cox analysis of *GPR37* expression, grade, histological type, *CDKN2A/B* homozygous deletion (homdel), radiation therapy, age, and gender for OS (**A**, **B**) and DSS (**C**, **D**). Abbreviations: OS: overall survival; DSS: disease specific survival; HR: hazard ratio.

Univariate Cox regression analysis of *GPR37* expression, grade, histological type, *CDKN2A/B* homozygous deletion, age, gender and primary therapy outcome using TCGA data indicated that *GPR37* was significantly correlated with both OS (HR 1.851, 95% CI = 1.450, 2.362, *p* < 0.001) and DSS (HR 2,031, 95% CI = 1.565, 2.637, *p* < 0.001). In addition, multivariate analysis identified *GPR37* as an independent risk factor for OS (HR 1.771, 95% CI = 1.180, 2.658, *p* = 0.006) and DSS (HR 1.834, 95% CI = 1.196, 2.813, *p* = 0.005, Supplementary Tables 3 and 4). Figure 4 shows a summary of the findings.

### Co-expressed genes of *GPR37* and GSEA

**Figure 5:**
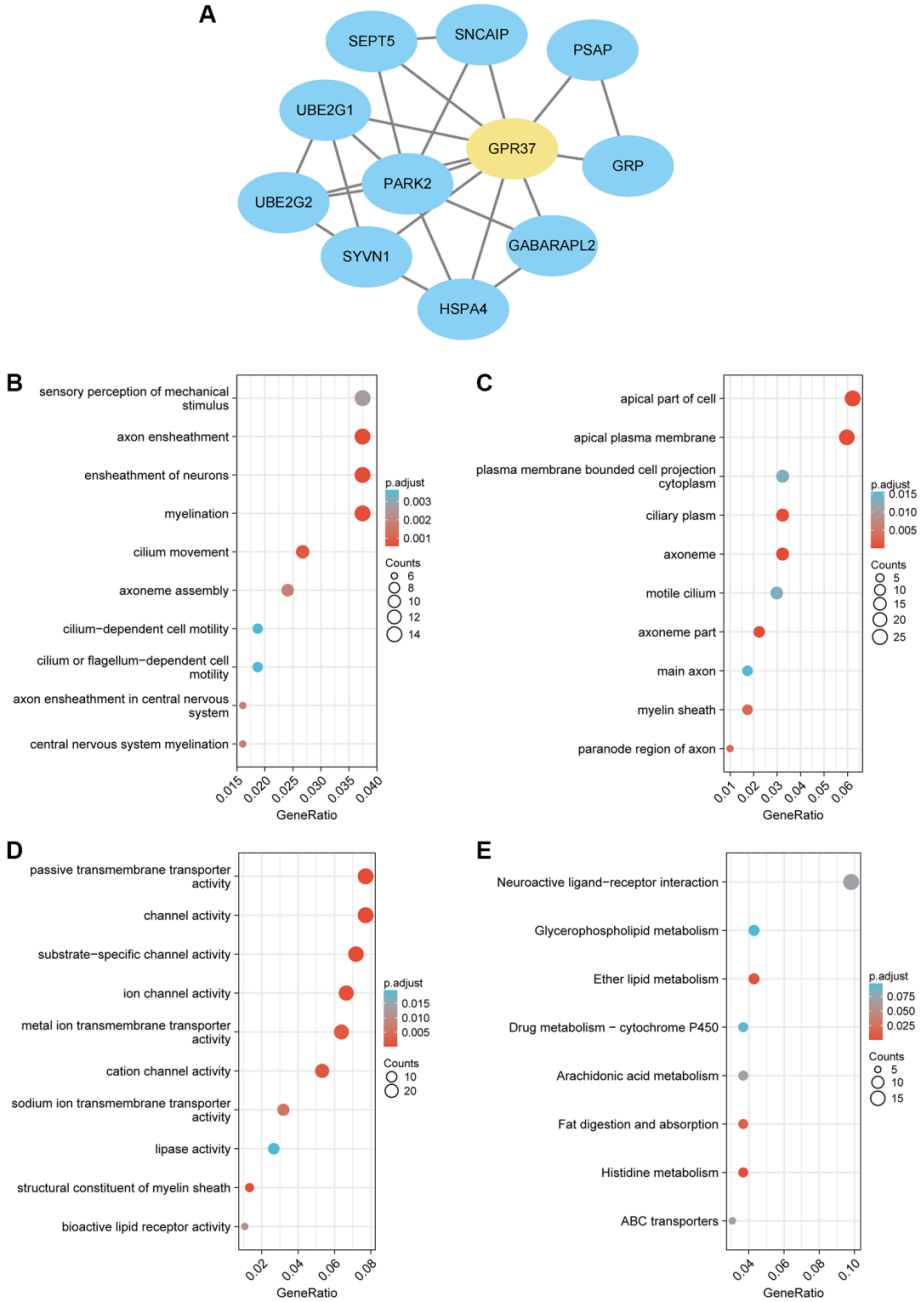
(**A**) PPI network of *GPR37*-related genes in glioma. (**B**–**E**) Gene set enrichment analysis based on GO analysis including BP, CC and MF terms, and KEGG pathway analysis for all linked hub genes of *GPR37* in glioma.

To explore the role played by *GPR37* in the development and progression of glioma, we screened for the co-expressed genes using the LinkedOmics database. The heatmaps of the top 50 positively and negatively *GPR37*-linked genes are shown in Supplementary Figure 1A, 1B. A PPI network of *GPR37* was constructed, and the top ten hub genes were *PARK2*, *UBE2G2*, *UBE2G1*, *SEPT5*, *SNCAIP*, *PSAP*, *GRP*, *GABARAPL2*, *HSPA4* and *SYVN1* (Figure 5A). GO enrichment analysis further showed that the hub genes were significantly associated with sensory perception of mechanical stimulus, axon ensheathment, ensheathment of neurons, ciliary movement and other BP terms, whereas apical part of cell, apical plasma membrane, cilium plasm, axoneme etc., were the enriched CC terms, and passive transmembrane transporter activity, channel activity, substrate−specific channel activity etc., were the enriched MF terms. In addition, KEGG analysis revealed a significant association with ether lipid metabolism, fat digestion and absorption, and histidine metabolism pathways (Figure 5B–5E).

**Figure 6:**
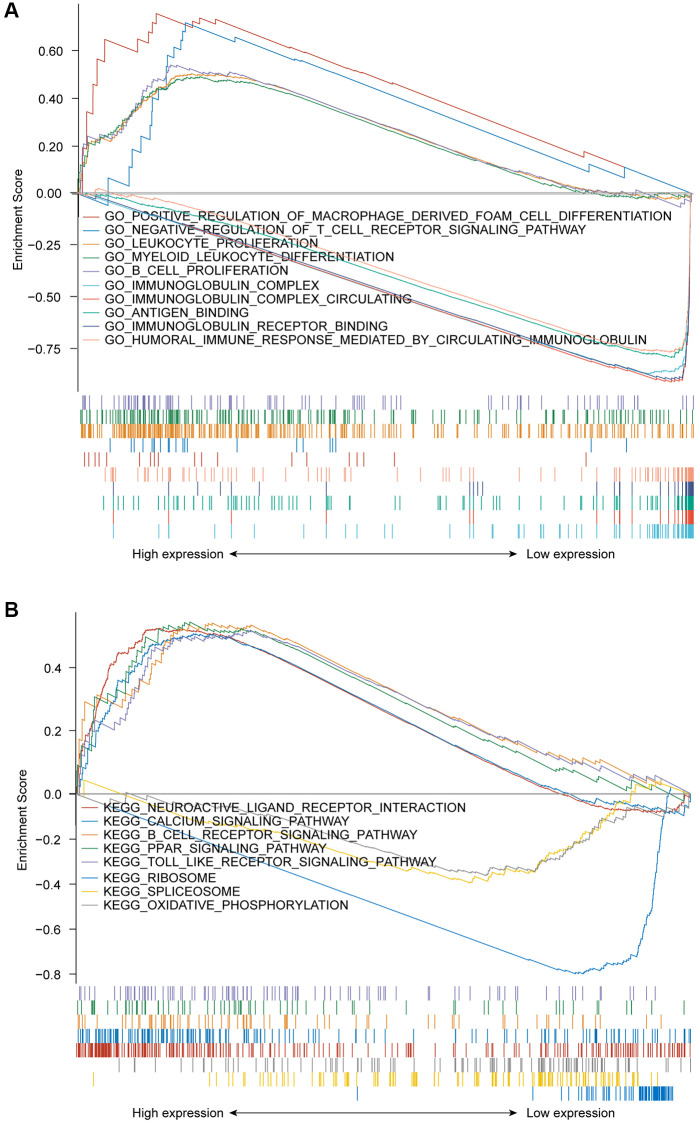
(**A**) GSEA results showing differential enrichment of GO terms as a function of *GPR37* expression. Top 5 GO terms for *GPR37*^high^- positive regulation of macrophage derived foam cell differentiation, negative regulation of T cell receptor signaling pathway, leukocyte proliferation, B cell proliferation and myeloid leukocyte differentiation. Top 5 GO terms for *GPR37*^low^- immunoglobulin complex, immunoglobulin complex circulating, antigen binding, immunoglobulin receptor binding and humoral immune response mediated by circulating immunoglobulin. (**B**) GSEA results showing differential enrichment of KEGG pathways as a function of *GPR37*. Top 5 KEGG pathways for *GPR37*^high^-neuroactive ligand receptor interaction, calcium signaling pathway, B cell receptor signaling pathway, PPAR signaling pathway and toll like receptor signaling pathway. Two KEGG pathways in *GPR37*^low^- ribosome, spliceosome and oxidative phosphorylation. All results of GSEA were based on NES, adjusted *P* value and FDR value. GSEA, gene set enrichment analysis.

GSEA was used to discriminate between the *GPR37*^high^ and *GPR37*^low^ glioma populations (adjust *P* value < 0.05, FDR < 0.05). The *GPR37*^high^ phenotype a significant enrichment of GO terms for positive regulation of macrophage derived foam cell differentiation, negative regulation of T cell receptor signaling pathway, leukocyte proliferation, B cell proliferation and myeloid leukocyte differentiation, whereas immunoglobulin complex, immunoglobulin complex circulating, antigen binding, immunoglobulin receptor binding and humoral immune response mediated by circulating immunoglobulin were significantly enriched in the *GPR37*^low^ phenotype (Figure 6A). Neuroactive ligand receptor interaction, calcium signaling pathway, B cell receptor signaling pathway, PPAR signaling pathway, and toll like receptor signaling pathway were the top 5 KEGG pathways in the *GPR37*^high^ groups, whereas the *GPR37*^low^ phenotype was associated with ribosome, spliceosome and oxidative phosphorylation pathways (Figure 6B). The GO and KEGG components are summarized in Supplementary Table 5. These results indicated that *GPR37* is involved in the development and progression of glioma.

### Tumor infiltration analysis

**Figure 7:**
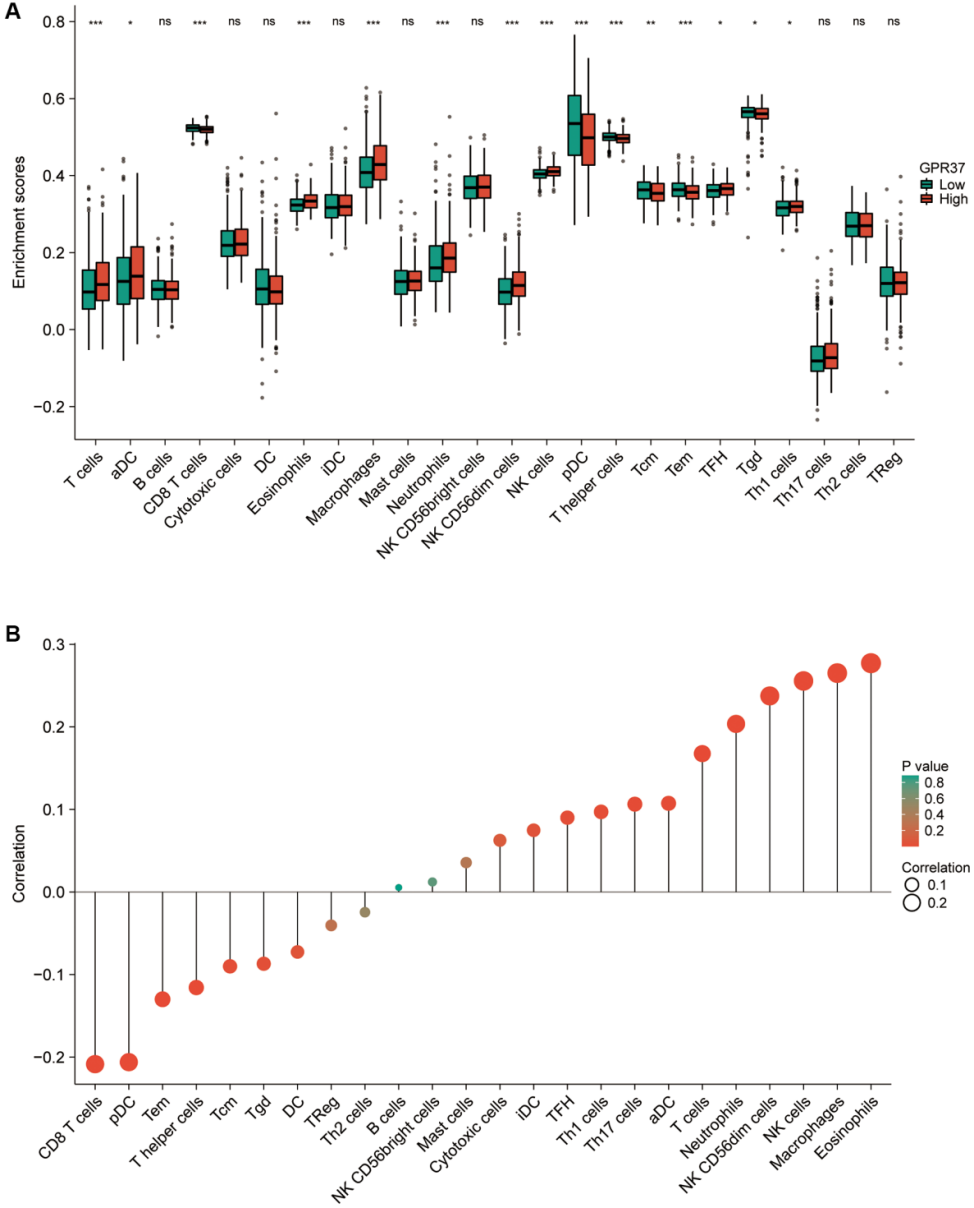
(**A**) Correlation between *GPR37* expression and 24 tumor-infiltrating immune cell types. (**B**) Eosinophils, Macrophages, NK cells, Neutrophils and T cells were positively connected with *GPR37* expression, while CD8 T cells, pDCs, Tem, T helper cells, Tcm, and Tgd were negatively correlated. ns, *p* ≥ 0.05; ^*^p < 0.05; ^**^p < 0.01; ^***^p < 0.001.

**Figure 8:**
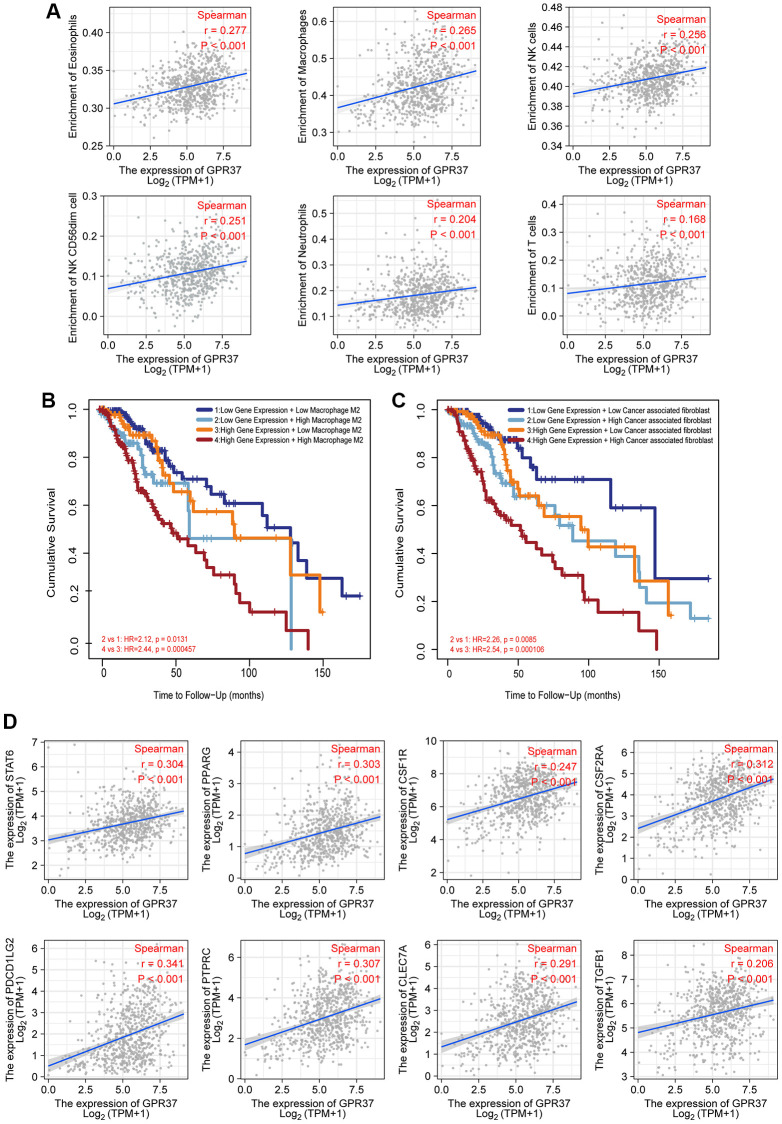
**Correlation between immunological infiltrates and *GPR37* expression.** (**A**) Eosinophils, Macrophages, NK cells, NK CD56^dim^ cells, Neutrophils, and T cells were all positively linked with *GPR37* expression. (**B**, **C**) Infiltration of M2 macrophages and cancer-associated fibroblasts were associated with poor outcome. (**D**) The M2 markers were positively linked with *GPR37* expression.

Tumor-infiltrating lymphocytes are reliable indicators of cancer survival. Therefore, we also analyzed the correlation between *GPR37* expression in glioma and immune infiltration. As shown in Figure 7A, *GPR37* expression correlated significantly with the infiltration of T cells, CD8 T cells, eosinophils, macrophages, neutrophils, NK CD56^dim^ cells, NK cells, plasmacytoid DCs (pDCs), T helper cells, T effector memory (Tem) cells (*P* < 0.001), T central memory (Tcm) cells (*P* < 0.01), T follicular helper (Tfh) cells, tgd, Th1 cells and activated DCs (aDCs) (*P* < 0.05). On the other hand, no significant correlation was seen with B cells, cytotoxic cells, DCs, immature DCs (iDCs), mast cells, NK CD56^bright^ cells, Th17 cells, Th2 cells and Treg cells. As shown in Figure 7B, *GPR37* was positively associated with infiltration levels of Eosinophils (r = 0.277, *P* < 0.001, Figure 8A), Macrophages (r = 0.265, *P* < 0.001, Figure 8A), NK cells (r = 0.256, *P* < 0.001, Figure 8A), NK CD56^dim^ cells (r = 0.251, *P* < 0.001, Figure 8A), Neutrophils (r = 0.204, *P* < 0.001, Figure 8A), T cells (r = 0.168, *P* < 0.001, Figure 8A), and negatively with that of CD8 T cells, pDC, Tem, T Helper cells, Tcm, Tgd etc. Furthermore, high M2 macrophage infiltration along with high expression of *GPR37* portended poor prognosis (Figure 8B, HR = 2.44, *p* = 0.000457), as did higher infiltration of cancer associated fibroblasts (Figure 8C). In addition, the expression levels of most M2 macrophage markers, including *STAT6*, *PPARG*, *CSF1R*, *CSF2RA*, *PDCD1LG2*, *PTPRC*, *CLEC7A*, *and TGF-1*, were positively correlated with *GPR37* (Figure 8D). Thus, *GPR37* may influence macrophage polarization in glioma.

### Correlation between immune checkpoints and GPR37 expression

**Figure 9:**
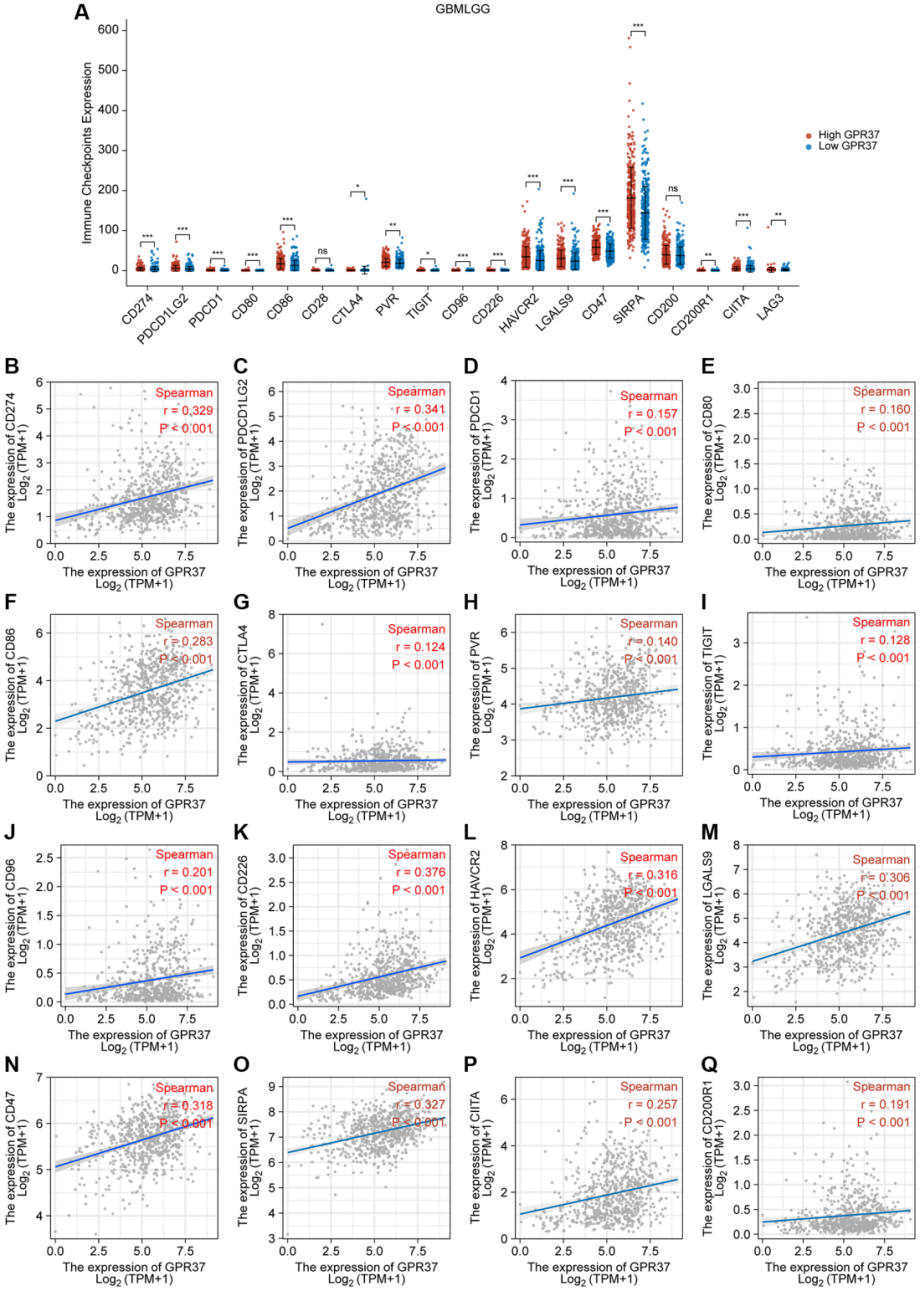
**Correlation between *GPR37* expression levels and common immune checkpoints (ICPs).** (**A**) Graph showing expression of ICPs in the *GPR37*^high^ (red) and *GPR37*^low^ (blue) groups. (**B**–**Q**) Spearman correlation coefficients for the association between the expression levels of *GPR37* and ICPs. ^*^P < 0.05, ^**^P < 0.01, ^***^P < 0.001.

We examined the relationship between *GPR37* levels and those of common immune checkpoints (ICPs) to determine the possible impact of *GPR37* expression on the response to immunotherapy. The *GPR37*^high^ group had high levels of *CD274*, *PDCD1LG2*, *PDCD1*, *CD80*, *CD86*, *CD28*, *CTLA4*, *PVR*, *TIGIT*, *CD96*, *CD226*, *HAVCR2*, *LGALS9*, *CD47*, *SIPRA*, *CD200*, *CD200R1*, *CIITA*, and *LAG3* expression (Figure 9A). Furthermore, TCGA-based analyses revealed a positive relationship between *GPR37* expression and *CD274*, *PDCD1LG2*, *PDCD1*, *CD80*, *CD86*, *CTLA4*, *PVR*, *TIGIT*, *CD96*, *CD226*, *HAVCR2*, *LGALS9*, *CD47*, *SIPRA*, *CD200*, *CD200R1*, *CIITA*, and *LAG3* expression (Figure 9B–9Q). These higher ICP levels suggested that patients with strong *GPR37* expression would have a better immunotherapy response.

### GPR37 DNA methylation analysis

**Figure 10:**
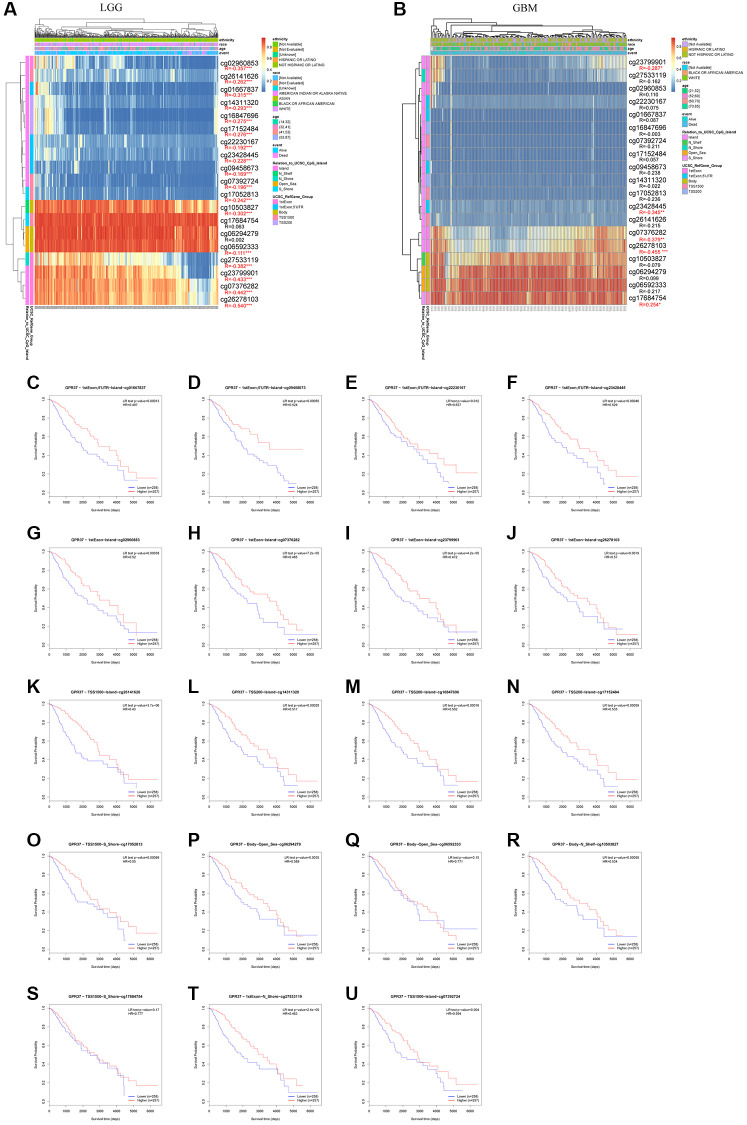
***GPR37* promoter methylation in glioma.** (**A**, **B**) Waterfall plots showing the correlation methylation level of *GPR37* promoter and gene expression. (**C**–**U**) Survival curves as a function of methylation sites. ^*^P < 0.05, ^**^P < 0.01, ^***^P < 0.001.

Since DNA methylation is a critical epigenetic modification that is associated with tumor progression, we also screened for the methylated sites in *GPR37* and analyzed the correlation between *GPR37* methylation and expression in glioma. As shown in Figure 10A, 10B, there was a significant negative correlation between the extent of DNA methylation in *GPR37* and its expression levels in LGG, and to a lesser extent in GBM. In addition, hypomethylation at cg02960853, cg26141626, cg01667837, cg14311320, cg16847696, cg17152484, cg22230167, cg23428445, cg09458673, cg07392724, cg17052813, cg10503827, cg06592333, cg27533119, cg23799901, cg07376282 and cg26278103 in the *GPR37* promoter was linked to poor prognosis (Figure 10C–10U). These findings were in line with our earlier study.

## DISCUSSION

GPCRs are the largest family of cell surface proteins involved in signal transduction, and key participants in tumor growth and metastasis [32]. Aberrant expression and function of GPCRs in tumor cells have been linked to autonomous proliferation, immune escape, increased metabolism, and invasion and metastasis to other tissues [33]. Although the involvement of *GPR37* in the growth and prognosis of several malignancies has been partially validated, its role in glioma remains unknown [34–36]. Through bioinformatics analysis, we found that *GPR37* was noticeably up-regulated in glioma tissues, and associated with poor prognosis. In addition, we also identified *GPR37* as an independent prognostic factor for both OS and DSS in glioma.

PPI network analysis further revealed that *GPR37* interacts with *PARK2*, *UBE2G2*, *UBE2G1*, *SEPT5*, *SNCAIP*, *PSAP*, *GRP*, *GABARAPL2*, *HSPA4* and *SYVN1*, which have been linked to tumor development in previous studies [37–44]. *GPR37* was previously identified as the receptor of Parkin, an E3 ubiquitin ligase encoded by *PARK2*, involved in ubiquitination and proteasome mediated degradation/clearance of misfolding proteins, which is closely related to tumor development [45–47]. Furthermore, one of the characteristics of *GPR37* is that it has an abnormally long N-terminal [48]. It has been proved that N-terminal truncation will make the surface transport of *GPR37* more efficient, resulting in enhanced expression of *GPR37* [49]. During the transport of *GPR37* protein from the endoplasmic reticulum (ER) to the cell membrane, metalloproteinases (MPs) can process the N-terminal of *GPR37*, so that *GPR37* can be transformed from precursor form to mature complete glycosylation form and stably exist in the cell membrane in the cleaved form [50]. In our research, GO enrichment analysis showed that *GPR37* is enriched in apical part of cell, apical plasma membrane, cilium plasm, axoneme etc., has molecular function of passive transmembrane transporter activity, channel activity, substrate−specific channel activity etc., and participates in biological processes such as sensory perception of mechanical stimulus, axon ensheathment, ensheathment of neurons, ciliary movement and so on. In addition to these, KEGG analysis revealed that *GPR37* may be involved in regulating ether lipid metabolism, fat digestion and absorption, and histidine metabolism pathways. Ether lipid biosynthesis is unique to the peroxisome and is regulated by the peroxisome proliferator activated receptor (PPAR) [51–54]. PPAR-γ is the major receptor in the central nervous system (CNS) and is generally expressed at a low level [55]. Nwankwo and Khoo et al. discovered that PPAR expression was significantly higher in gliomas compared to normal astrocytes, and was associated with a poor prognosis [56, 57]. PPAR-γ primarily regulates gene expression at the transcriptional level, and is in turn regulated by transcription factors, microRNAs and kinases, thus affecting stemness and malignant transformation [58]. We found that the highly expressed *GPR37* was enriched in the PPAR signaling pathway, which is consistent with previous findings. In summary, *GPR37* may increase the incidence and development of glioma by regulating the PPAR pathway, which requires more investigation.

The tumor immune microenvironment consists of various immune and inflammatory cells that play an important role in tumor development and progression [59]. In this study, we confirmed that the abnormal expression of *GPR37* is related to the increased infiltration of eosinophils, macrophages, NK CD56^dim^ cells, NK cells, neutrophils and T cells etc. A number of growth factors and cytokines can be released by tumor associated macrophages (TAMs), which are drawn into the glioma environment, have immunological capabilities, and are able to react to the growth factors produced by cancer cells [60]. TAMs encourage tumor migration, survival, and proliferation in this way. *GPR37* was significantly associated with the infiltration of macrophages, which are the most prominent inflammatory cells in tumor tissues [61]. The tumor-associated macrophages (TAMs) of the M1 phenotype promote inflammation, and inhibit tumor growth and invasion. The M2 macrophages on the other hand are anti-inflammatory and pro-tumorigenic [62, 63]. Glioma cells release a range of chemokines (CSF-1, MCP-1 and others) to recruit and activate TAMs, which then secrete the anti-inflammatory cytokines *IL-10* and *TGFB1* [64]. Furthermore, activation of *GPR37* in macrophages facilitates phagocytosis and the regression of inflammatory pain [65]. Bang and Qu et al. found that *GPR37*, the receptor of specialized pro-resolving mediators (SPMs), can bind to neuroprotectin D1 (*NPD1*) to inhibit the proinflammatory cytokine IL-1 and increase production of IL-10 and *TGFB1*. This polarizes the macrophages to the M2 phenotype and relieves inflammatory pain [66, 67]. Consistent with these reports, we found that the expression of *GPR37* was positively correlated with M2-like TAM markers, and higher proportion of M2 macrophages predicted worse prognosis in the *GPR37*^high^ patients. Thus, *GPR37* maybe contribute to glioma progression by recruiting TAMs and promoting M2 polarization.

Immune checkpoint inhibitors (ICIs) can effectively eliminate tumor cells by activating the anti-tumor immune responses [68, 69]. ICPs inhibit T cell function and survival by preventing antigen presentation, and overactivation of ICPs is a common strategy used by the tumor cells to avoid immune detection [70, 71]. The microenvironment of the CNS was long considered immunosuppressive due to the presence of the blood-brain barrier (BBB), and thus a major impediment to the use of ICIs in the treatment of gliomas [72]. Although this surmise has been challenged in recent years, a huge proportion of glioma patients fail to respond to ICIs. Therefore, it is crucial to identify the biomarkers of gliomas that may affect patient responsiveness to ICIs therapy [73]. We found that *GPR37* was positively correlated with several ICPs such as *CD274*, *PDCD1LG2*, *PDCD1*, *CD80*, *CD86*, *CTLA4*, *PVR*, *TIGIT*, *CD96*, *CD226*, *HAVCR2*, *LGALS9*, *CD47*, *SIPRA*, *CD200*, *CD200R1*, *CIITA* and *LAG3*, indicating that *GPR37* is a promising immune-related gene that can influence immunotherapy response in glioma patients.

DNA methylation is one of the variables of tumor growth [74, 75]. Promoter hypermethylation have been linked to reduced transcription or gene silencing, whereas hypermethylation of promoters leads to increased gene expression [76, 77]. In line with previous reports, we found that hypomethylation of the *GPR37* promoter was associated with increased expression, and this result was more obvious in LGG than in GBM. Furthermore, LGG patients with hypermethylated promoter regions showed better prognosis. Therefore, the impact of specific *GPR37* methylation sites on gene expression and mortality, particularly in LGG patients, needs further investigation.

## CONCLUSIONS

*GPR37* is frequently overexpressed in glioma and is an independent prognostic predictor. It is involved in peroxisome control via the PPAR pathway, and is also associated with the infiltration of M2 macrophages and other immune cells. What is more, the overexpression of *GPR37* in glioma may be due to DNA hypomethylation. Our findings provide new insights into the possible mechanisms of glioma progression, particularly in the context of the tumor immune environment, which can help develop individualized treatment.

## Supplementary Materials

Supplementary Figure 1

Supplementary Table 1

Supplementary Tables 2-5
