# Supplementary material for: GPR37 expression as a prognostic marker in gliomas: a bioinformatics-based analysis
Source: Aging (Albany NY). 2023 Oct 13;15(19):10146–67. doi: 10.18632/aging.205063 (PMC10599758; doi:10.18632/aging.205063)
Supplement: Supplementary Figure 1 [file aging-15-205063-s001.pdf]

SUPPLEMENTARY FIGURE

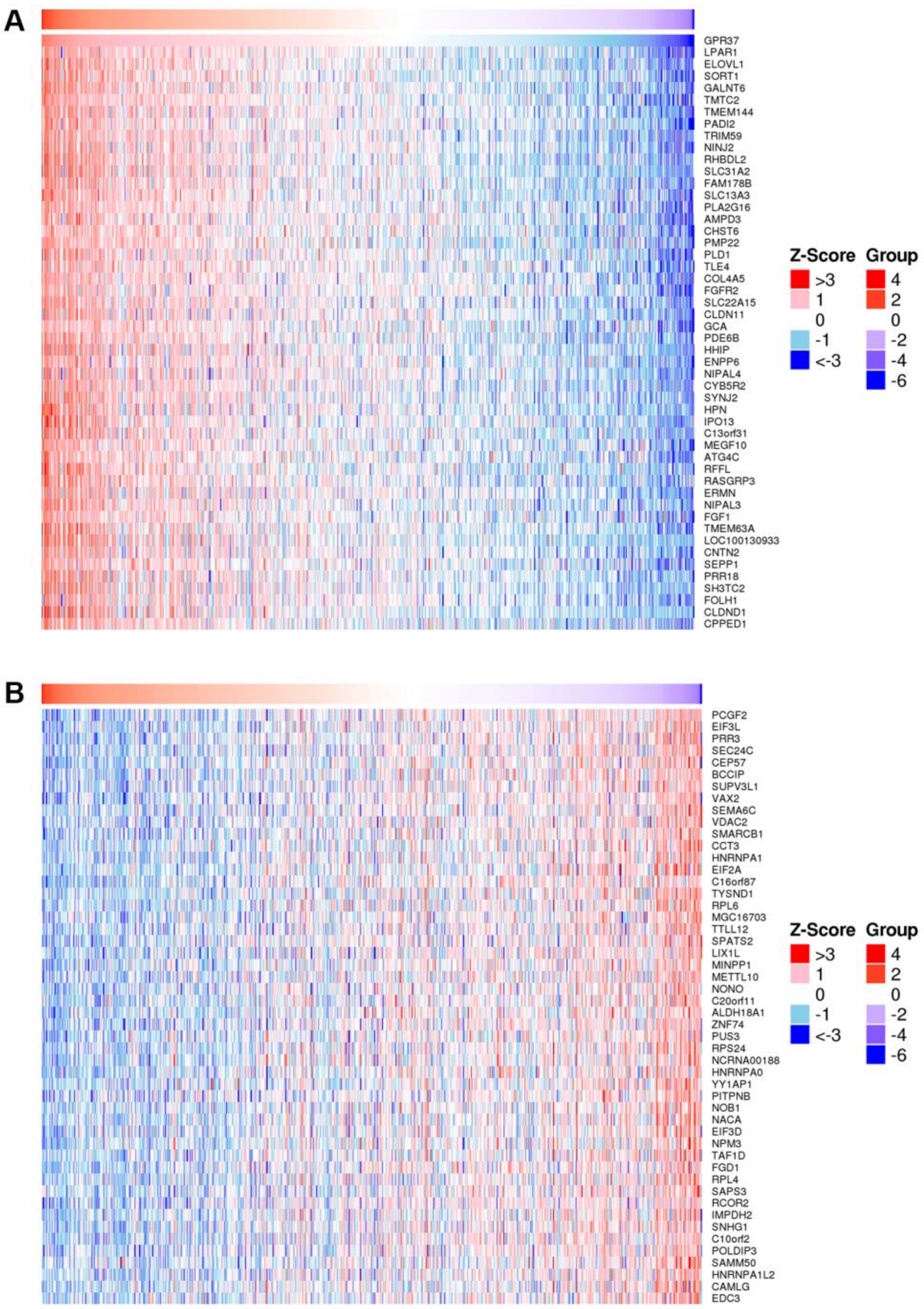

Supplementary Figure 1. Co-expression and gene set enrichment analysis in glioma. (**A**) Heatmap of the 50 negatively correlated genes of *GPR37*. (**B**) Heatmap of the top 50 positively correlated genes of *GPR37*.
