# Supplementary material for: GPR37 expression as a prognostic marker in gliomas: a bioinformatics-based analysis
Source: Aging (Albany NY). 2023 Oct 13;15(19):10146–67. doi: 10.18632/aging.205063 (PMC10599758; doi:10.18632/aging.205063)
Supplement: Supplementary Tables 2-5 [file aging-15-205063-s003.pdf]

SUPPLEMENTARY TABLES

Supplementary Table 2. The clinicopathological variables of 38 patients.

Characteristics	Total (*N*)
Grade, *n* (%)	
G2 & G3	9 (23.7%)
G4	29 (76.3%)
Histological type, *n* (%)	
Oligodendroglioma, IDH mut, 1p/19q codel	4 (10.5%)
Astrocytoma, IDH mut	10 (26.3%)
Glioblastoma	24 (63.2%)
Gender, *n* (%)	
Male	22 (57.9%)
Female	16 (42.1%)
Age, *n* (%)	
≤60	27 (71.1%)
>60	11 (28.9%)

Supplementary Table 3. Cox analysis of GPR37 expression and other clinicopathological variables for OS (univariate and multivariate).

Characteristics	Total (*N*)	HR (95% CI) Univariate analysis (OS)	*P* value Univariate analysis	HR (95% CI) Multivariate analysis (OS)	*P* value Multivariate analysis
GPR37	698		<0.001		
Low	351	Reference		Reference	
High	347	1.851 (1.450–2.362)	<0.001	1.771 (1.180–2.658)	0.006
Grade	695		<0.001		
G4	284	Reference		Reference	
G2 & G3	411	0.116 (0.088–0.153)	<0.001	2.482 (0.962–6.405)	0.060
Histological Type	688		<0.001		
Glioblastoma, IDH wildtype	246	Reference		Reference	
Oligodendroglioma, IDH mutation, 1p/19q-codel	171	0.081 (0.052–0.125)	<0.001	0.130 (0.048–0.354)	<0.001
Astroctyoma, IDH mutation	271	0.137 (0.103–0.184)	<0.001	0.211 (0.090–0.494)	<0.001
CDKN2A/B homdel	698		<0.001		
Non-homdel	549	Reference		Reference	
Homdel	149	5.332 (4.123–6.896)	<0.001	4.770 (2.617–8.694)	<0.001
Age	698		<0.001		
≤60	555	Reference		Reference	
>60	143	4.696 (3.620–6.093)	<0.001	4.189 (2.639–6.650)	<0.001
Gender	698		0.071		
Male	401	Reference			
Female	297	0.800 (0.627–1.021)	0.073		
Primary therapy outcome	464		<0.001		
PR & CR	204	Reference		Reference	
PD & SD	260	4.868 (2.783–8.514)	<0.001	4.000 (2.268–7.055)	<0.001

Supplementary Table 4. Cox analysis of GPR37 expression and other clinicopathological variables for DSS (univariate and multivariate).

Characteristics	Total (*N*)	HR (95% CI) Univariate analysis (DSS)	*P* value Univariate analysis	HR (95% CI) Multivariate analysis (DSS)	*P* value Multivariate analysis
GPR37	677		<0.001		
Low	339	Reference		Reference	
High	338	2.031 (1.565–2.637)	<0.001	1.834 (1.196–2.813)	0.005
Grade	674		<0.001		
G4	270	Reference		Reference	
G2 & G3	404	0.105 (0.078–0.141)	<0.001	2.427 (0.926–6.364)	0.071
Histological Type	667		<0.001		
Glioblastoma, IDH wildtype	232	Reference		Reference	
Oligodendroglioma, IDH mutation,1p/19q-codel	170	0.071 (0.044–0.115)	<0.001	0.112 (0.040–0.315)	<0.001
Astroctyoma, IDH mutation	265	0.133 (0.098–0.182)	<0.001	0.199 (0.085–0.468)	<0.001
CDKN2A/B homdel	677		<0.001		
Non-homdel	537	Reference		Reference	
Homdel	140	5.457 (4.167–7.147)	<0.001	4.716 (2.573–8.646)	<0.001
Age	677		<0.001		
≤60	544	Reference		Reference	
>60	133	4.528 (3.430–5.978)	<0.001	3.815 (2.352–6.190)	<0.001
Gender	677		0.104		
Male	388	Reference			
Female	289	0.809 (0.625–1.047)	0.107		
Primary therapy outcome	460		<0.001		
PR & CR	204	Reference		Reference	
PD & SD	256	5.486 (3.009–10.001)	<0.001	4.442 (2.412–8.182)	<0.001

Supplementary Table 5. Gene enrichment study based on the phenotypes of high and low GPR37 expression.

Gene set name	NES
High expression	
GO_NEGATIVE_REGULATION_OF_T_CELL_RECEPTOR_SIGNALING_PATHWAY	1.766
GO_REGULATION_OF_MACROPHAGE_DERIVED_FOAM_CELL_DIFFERENTIATION	1.737
GO_LEUKOCYTE_PROLIFERATION	1.69
GO_B_CELL_PROLIFERATION	1.668
GO_MYELOID_LEUKOCYTE_DIFFERENTIATION	1.612
KEGG_NEUROACTIVE_LIGAND_RECEPTOR_INTERACTION	1.754
KEGG_CALCIUM_SIGNALING_PATHWAY	1.654
KEGG_B_CELL_RECEPTOR_SIGNALING_PATHWAY	1.621
KEGG_PPAR_SIGNALING_PATHWAY	1.612
KEGG_TOLL_LIKE_RECEPTOR_SIGNALING_PATHWAY	1.608
Low expression	
GO_HUMORAL_IMMUNE_RESPONSE_MEDIATED_BY_CIRCULATING_IMMUNOGLOBULIN	−3.223
GO_IMMUNOGLOBULIN_RECEPTOR_BINDING	−3.357

GO_ANTIGEN_BINDING	−3.36
GO_IMMUNOGLOBULIN_COMPLEX_CIRCULATING	−3.37
GO_IMMUNOGLOBULIN_COMPLEX	−3.678
KEGG_OXIDATIVE_PHOSPHORYLATION	−1.469
KEGG_SPLICEOSOME	−1.626
KEGG_RIBOSOME	−3.092
